# Exploratory factor analysis of items for evaluating healthy lifestyles related to mental health among undergraduate students.

**DOI:** 10.51866/oa.448

**Published:** 2023-10-21

**Authors:** Zarizi Ab Rahman, Badrul Isa, Mohd Sazili Shahibi, Muhammad Irsyad Mansor

**Affiliations:** 1 BEd, MEd, PhD, Faculty of Education, Universiti Teknologi MARA, UiTM Puncak Alam Campus, Puncak Alam, Selangor, Malaysia. Email: zarizi@uitm.edu.my; 2 BEd, MEd, PhD, Faculty of Education, Universiti Teknologi MARA, UiTM Puncak Alam Campus, Puncak Alam, Selangor, Malaysia.; 3 BMassComm, MSc, PhD, College of Computing, Informatic and Media, Al-Khawarizmi Building, Universiti Teknologi MARA, Shah Alam, Selangor, Malaysia.; 4 BEd, Faculty of Education, Universiti Teknologi MARA, UiTM Puncak Alam Campus, Puncak Alam, Selangor, Malaysia.

**Keywords:** Healthy lifestyles, Mental health, Intervention, Well-being

## Abstract

**Introduction:**

A healthy lifestyle is pivotal for improving mental health. As the concept of a healthy lifestyle is comprehensive, there is a need to prioritise components related to mental health in planning proper mental health interventions. In this regard, physical activity, diet, sleep quality, substance abuse and social support have been identified to enhance mental health. It is necessary to develop a valid scale for assessing healthy lifestyles related to mental health. Thus, this study aimed to adapt and validate an existing scale to evaluate healthy lifestyles related to mental health.

**Method:**

This study included 177 (men: n=73, women: n=104) participants from University Teknologi MARA, Puncak Alam, Selangor. The data were presented using descriptive statistics and subjected to an exploratory factor analysis.

**Results:**

The 22 scale items evaluated were valid and reliable in assessing the five components of healthy lifestyles related to mental health. The total variance explained for measuring the construct was 68.610%. The Cronbachs alpha value for the five components ranged from 0.784 to 0.903.

**Conclusion:**

The adapted scale is acceptable and reliable in evaluating healthy lifestyles related to mental health within the target population. Thus, it can be used to assess significant components of healthy lifestyles to promote mental health. Accordingly, relevant authorities can formulate the best strategies to enhance mental health.

## Introduction

Undergraduate students are susceptible to mental health problems due to academic stress and social issues. It has been reported that 49% and 41% of health sciences and accountancy students at Universiti Teknologi MARA (UiTM), Puncak Alam, Selangor, experience anxiety, respectively,^1^ while students in Shah Alam experience severe depression, anxiety and stress.^2^ Healthy lifestyle components, including relationships, social support, healthy diet, smoking, alcohol consumption, sleep quality and physical exercise, could influence mental health bidirectionally.^3,4^ Promoting healthy lifestyles can enhance mental health, and interventions should consider protective and modifiable factors to ensure the effectiveness of behavioural changes.^5^ Appropriate instruments are necessary to assess protective factors.

Many studies have emphasised the role of physical activity, diet, substance abuse, sleep quality and social support in mental health. Thus, there is a need to explore the benefits of a healthy lifestyle owing to the potential effect on mental health. Physical activity comprises various movement activities, including any skeletal muscle-driven body movements requiring the use of energy beyond the resting levels.^6^ Past studies have shown that physical activity enhances mental health, as it boosts mood and self-esteem and reduces stress. Engaging in 150 min of moderate-to-vigorous physical activity weekly could reduce symptoms of depression.^7^ Moreover, 60-min aerobic activities three to five times a week are recommended for optimal well-being.

A healthy diet consists of food that positively impacts health or, at the very least, has no adverse health effects.^8^ According to the World Health Organization, inadequate nutrient intake increases the risk of diseases such as hypertension.^9^ A diet comprising fruits, vegetables, fish, legumes, lean meats, olive oil and whole grains can improve mental health and reduce the risk of depression.^9^ In contrast, a diet consisting of processed, fried and sugary foods is linked to depression and anxiety, especially among adolescents. Undergraduate students generally consume unhealthy diets, which could impact their mental health. Unhealthy dietary habits such as skipping breakfast, reducing fruit intake, snacking too frequently and eating fried and fast food could affect the body’s oxidative stress indicators, immune system markers and inflammatory factors and subsequently disrupt one’s mental health.^10^

Substance abuse refers to repeated or continuous use of addictive substances such as alcohol, tobacco, and drugs.^11^ It is commonly linked to health issues, including mental health problems. Studies have found that university students who smoke and consume alcohol as stress coping mechanisms are more susceptible to depression and anxiety.^12^ Hence, there is a high prevalence of mental health issues among students who smoke and drink alcohol.^13^ Numerous studies have confirmed the adverse impacts of substance abuse on one’s health. For instance, binge drinking can lead to a high glucose level and blood pressure, while smoking is linked to adverse lipid and lipoprotein profiles. These conditions could subsequently lead to depression.^14^

Sleep quality is a crucial aspect of quality of life, as it denotes one’s satisfaction with all aspects of sleeping experience.^15^ Research has shown that sleep quality can significantly affect overall health. Sufficient high-quality sleep can improve psychological well-being and prevent mental health issues.^16^ Conversely, low-quality sleep can lead to hypertension, depression, and anxiety. Academic and psychological pressures and extracurricular activities are probable causes of poor sleep quality among undergraduate students.^17^ In this regard, adolescents need at least 7 h of sleep each night to ensure a healthy lifestyle.

Social support includes access to resources and aids such as coping mechanisms, resource sharing and personality attributes. It is an essential component of healthy lifestyles and mental health.^18^ Social support significantly improves health, preventing unhealthy behaviours and mental health issues, especially among adolescents.^18^ Youths with adequate social support typically have positive interactions with others and, subsequently, better mental health. For students, social support includes relationships with teachers and peers, who significantly impact their behaviour. Undergraduate students often obtain essential social support and mental health benefits from their housemates or good friends in their campus. This shows the importance of friendship in providing social support. Friends provide optimal social support through shared interests, secure relationships, and peer recognition.^19^ Hence, social support is crucial for maintaining emotional well-being, effectively protecting against mental health issues. In this regard, having close friends and family creates a sense of safety and support.

Previous studies^7,9,12,13,16,18^ have indicated the benefits of a healthy lifestyle on mental health. Thus, this study aimed to adapt and validate a healthy lifestyle questionnaire to ensure its suitability in the context of mental health among the target population. An instrument’s validity and reliability are the most important factors to ensure accuracy and data usefulness. Owing to population disparities, questionnaires used to evaluate healthy lifestyles are often inappropriate. For instance, the validity of a quality of life questionnaire could be compromised if used in contexts other than where it is initially intended.^20^ Therefore, a questionnaire should be in line with the characteristics of the study population, such as age, sex, cultural context and educational background, and the purpose of the study.^21^

## Methods

### Participants

In this study, a survey was conducted among first- to fourth-year undergraduate students aged 19–23 years at UiTM, Puncak Alam. The lottery method was used to select a random sample from the sampling frame. Students from six faculties and two colleges at UiTM, Puncak Alam, who could read and understand Malay or English were included. The sample consisted of 177 respondents selected randomly; this size was considered sufficient in generalising the results to the population and appropriate for an exploratory factor analysis (EFA). Most scholars agree that the number of participants should be at least 100.^22^

### Instrument

The study instrument was adapted from the Adolescent Healthy Lifestyle Questionnaire (AHLQ),^23^ one of the questionnaires used in populations in Klang Valley. The AHLQ has a similar goal and target population to the present study. Hence, this questionnaire is the most suitable questionnaire that can be adapted to the target population. The original AHLQ was developed to evaluate healthy lifestyles among adolescents aged 13–17 years in Klang Valley. The AHLQ has 64 items across various healthy lifestyle components. Since the AHLQ targets school students, some items and components might not be relevant to this study. Hence, the original instrument was slightly modified to suit undergraduate students at UiTM, Puncak Alam.

The items were refined, and the constructs were summarised, focusing on the relationship of diet, physical activity, social support, sleep quality and substance abuse with mental health. The related constructs, such as social health and interpersonal relationships, were combined with social support, and the terms ‘teacher’ and ‘school’ were changed to ‘lecturer’ and ‘campus’, respectively. The first section assessed the respondents’ demographic background. The next section contained 25 items on the five constructs related to healthy lifestyles. The respondents were asked to rate their agreement on the statements using a 5-point Likert scale ranging from 1 (strongly disagree) to 5 (strongly agree). Five experts were consulted to ensure the questionnaire’s content validity based on the content clarity, relevance, and quality. According to Mohd Noah and Ahmad’s formula,^24^ a value of 0.80 indicates a good content validity.

### Ethical considerations

This study was approved by UiTM Research Ethics Committee under reference number ED/REC/F/10319. Informed consent was obtained from all participants.

## Results

A total of 177 students who fulfilled the inclusion criteria completed the questionnaire. In particular, the sample consisted of 104 (58.81%) female and 73 (41.2%) male first-to fourth-year students aged 19–23 years (Table 1).

**Table 1:** Demographic characteristics of the participants (N=177).

Characteristic	Semester	Frequency	Percentage
*Sex*			
Male		73	41.2
Female		104	58.8
Semester	2	105	59.3
	3	13	7.3
	4	23	13.0
	5	7	4.0
	6	11	6.2
	7	4	2.3
	8	14	7.9
*Faculty/college*			
Faculty of Education		32	18.1
Faculty of Health Science		16	9.0
Faculty of Pharmacy		9	5.1
College of Built Environment		21	11.9
Faculty of Hotel and Tourist Management		16	9.0
Faculty of Accountancy		16	9.0
College of Creative Art		12	6.8
Faculty of Business Management		55	31.1
		177	100.0

### Normality test

A normal distribution is a fundamental assumption for statistical analyses. The present study evaluated the skewness and kurtosis of the data. An absolute skewness value of >3 and a kurtosis value of >10 indicate a problem.25 Hence, the absolute skewness and kurtosis values should not exceed 3 and 10, respectively. Herein, the absolute skewness and kurtosis values of all items were within the acceptable ranges.

### EFA

This study adapted items from an existing questionnaire to suit the target population’s requirements. An EFA was used to re-evaluate each item to ensure applicability to the present study. Twenty-five items were analysed via principal component analysis extraction and varimax rotation. The factor loading for the study was set at a minimum of 0.50. The study extracted items with factor loadings of at least 0.50 and eliminated three items (SS23, SS24 and SS25) with factor loadings below 0.50. As shown in Table 2, the result of Bartlett’s test of sphericity was significant (P<0.05). Additionally, the sampling adequacy based on the Kaiser—Meyer—Olkin (KMO) value (0.857) was acceptable. A KMO value of 0.60–0.70 is considered adequate for an EFA.

**Table 2:** KMO and Bartlett’s test of sphericity.

KMO measure of sampling adequacy		0.857
Bartlett’s test of sphericity	Chi-square	2815.791
	df	300
	Sig.	0.000

KMO, Kaiser-Meyer-Olkin

### Components and total variance explained

Table 3 shows the five components evaluated in the EFA. All eigenvalues were greater than 1.0, ranging from 1.304 to 8.684. The variance explained for component 1 was 34.736%; component 2, 12.933%; component 3, 8.855%; component 4, 6.869%; and component 5, 5.216%. The total variance explained for the questionnaire construct was 68.610%, exceeding the minimum requirement of 60%.

**Table 3:** Total variance explained.

Component	Initial eigenvalue			Rotation sum of squared loadings		
	Total	% of variance	Cumulative %	Total	% of variance	Cumulative %
1	8.684	34.736	34.736	3.780	15.121	15.121
2	3.233	12.933	47.669	3.646	14.585	29.706
3	2.214	8.855	56.524	3.558	14.232	43.938
4	1.717	6.869	63.394	3.289	13.157	57.094
5	1.304	5.216	68.610	2.879	11.516	68.610

The screen plot in Figure 1 shows that five components emerged from the EFA. The EFA grouped the 25 items into five distinct components with certain numbers of items. The rotated component matrix shows the items grouped under each component.

**Figure:**
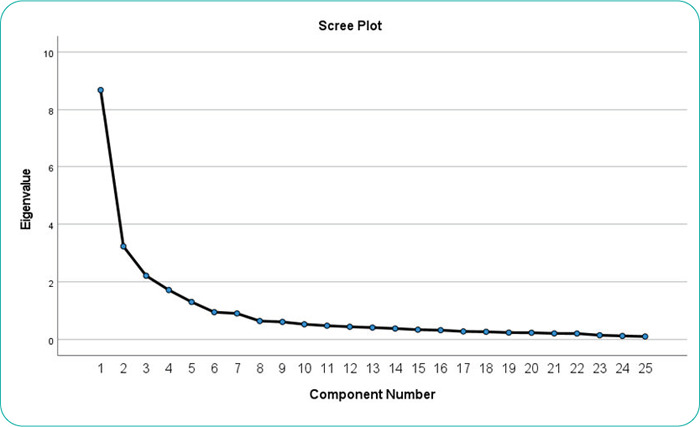


Table 4 lists the factor loadings for the five components in the EFA. The factor loading for each item was higher than 0.50, except for SS23 (‘I disagree with my lectures on many things’), SS24 (‘I disagree with my friends on many things’) and SS25 (‘I disagree with my parents on many things’). Twenty-two items were retained to measure the relationship between healthy lifestyles and mental health.

**Table 4:** Five components and items of the questionnaire on healthy lifestyles related to mental health.

	Rotated component matrix	Component				
		1	2	3	4	5
**Physical activity**						
PA16	I often do leisure activities in my free time. *Saya sering melakukan aktiviti riadah pada masa lapang*	0.793				
PA18	I exercise (e.g. jogging, cycling, swimming and playing soccer, netball or basketball) actively 30 min a day at least three times a week. *Saya bersenam (jogginglbermain bola sepaklberbasikall berenanglbola jaringlbola keranjang) dengan aktif 30 minit sehari sekurang-kurangnya 3 kali seminggu*	0.785				
PA17	Physical activity is part of my lifestyle. *Saya menjadikan aktiviti fizikal sebahagian daripada gaya hidup*	0.784				
PA14	I do physical activity when I am on campus. *Saya melakukan aktiviti fizikal ketika saya berada di kampus*	0.742				
PA15	I am actively involved in sports activities at campus. *Saya aktif melibatkan diri dalam aktiviti sukan di kampus*	0.718				
**Substance abuse**						
SA3	Smoking has a negative effect on me. *Merokok mendatangkan kesan buruk kepada saya*		0.849			
SA4	I stay away from smoking because it is one of the causes of health problems. *Saya menjauhi aktiviti merokok kerana ia merupakan salah satu punca masalah kesihatan*		0.836			
SA1	Drug use has a detrimental effect on me. *Penggunaan dadah mendatangkan kesan buruk kepada saya*		0.797			
SA5	I have never drunk alcoholic beverages. *Saya tidak pernah minum minuman memabukkan*		0.717			
SA2	Drinking intoxicating drinks has a bad effect on me. *Minum minuman memabukkan mendatangkan kesan buruk kepada saya*		0.558			
**Diet**						
HD9	I limit my intake of food high in fats. *Saya menghadkan pengambilan makanan tinggi lemak*			0.839		
HD10	I reduce my intake of fried food. *Saya mengurangkan pengambilan makanan bergoreng*			0.815		
HD8	I limit my intake of junk food. *Saya menghadkan pengambilan makanan ringan*			0.810		
HD6	I reduce my intake of sweetened beverages. *Saya mengurangkan pengambilan minuman yang manis*			0.668		
HD7	I reduce my intake of carbonated drinks. *Saya mengurangkan pengambilan minuman berkarbonat*			0.623		
**Sleep quality**						
SQ11	I have difficulty sleeping. *Saya mengalami kesukaran untuk tidur*				0.762	
SQ13	I find it difficult to sleep after being jerked awake at night. *Saya sukar melelapkan mata apabila terjaga pada waktu malam*				0.725	
SQ12	I often sleep late at night. *Saya sering tidur lewatpada waktu malam*				0.591	
**Social support**						
SS21	My peers help me when I have problems. *Rakan sebaya membantu saya apabila menghadapi masalah*					0.806
SS19	My peers care about me. *Rakan sebaya mengambil berat tentang saya*					0.769
SS20	I have fun spending time with my friends. *Saya seronok menghabiskan masa bersama rakan*					0.725
SS22	I share problems with my peers. *Saya meluahkan masalah kepada rakan sebaya*					0.711

Extraction method: Principal component analysis

Rotation method: Varimax with Kaiser normalisation

^a^Rotation converged in six iterations

### Internal reliability of the instrument

The Cronbach’s alpha value for each component of the scale was determined to evaluate its internal reliability. The values for the five components ranged from 0.784 to 0.903, exceeding the accepted alpha value of 0.65–0.80 for the human dimension. Generally, an alpha value of 0.76–0.95 is considered fairly high.^26^

## Discussion

This study aimed to adapt and validate the AHLQ^23^ to examine the relationship of physical activity, diet, substance abuse, sleep quality and social support with mental health. A few items needed to be revised owing to the different study population. An EFA was needed to identify the appropriate items, as the existing questionnaire targeted a different population. Hence, an EFA was performed to determine the number and nature of common factors explaining the correlation pattern between the items and the suitable number of factors needed to reproduce the item correlation matrix.^27^ Compared with a confirmatory factor analysis (CFA), an EFA allows researchers to find elements empirically unrelated to the intended construct and assess a questionnaire’s psychometric properties during the initial instrument development or adaptation phase to determine validity. In this study, the EFA revealed a five-factor structure with a high reliability for each factor (0.784–0.903). The scale’s total variance 68.610%. There were 22 items representing five vital components of a healthy lifestyle linked to mental health derived from the literature review.^9,11,15,16,18^ The results showed that the questionnaire had good validity, suggesting that it can be used for assessing healthy lifestyles related to mental health in the target population.

Regarding healthy lifestyles and mental health, the Simple Lifestyle Indicator Questionnaire^28^ contains 12 items under five constructs: diet, physical activity, smoking, stress, and alcohol consumption. All forms of substance abuse (e.g. alcohol, drugs or tobacco) should be considered. Other important aspects to consider for improving mental health are social support and sleep quality. Similar to the AHLQ, the Healthy Lifestyle Questionnaire^29^ for Ecuadorian University Students (EVS-EUE) has five constructs. However, the EVS-EUE does not include social support and other substance abuse linked to mental health.

The study provides evidence supporting the instrument’s validity and reliability. This suggests that the questionnaire is well suited for use among undergraduate students at UiTM, Puncak Alam. Employing an appropriate instrument is essential in the current context, as it enables the identification of the significant components of a healthy lifestyle that may help improve mental health. Accordingly, this aids in evaluating current policies and developing more targeted interventions or strategies to promote a healthy lifestyle and enhance mental health within the specified population. In this context, the promotion of a healthy lifestyle and mental health aligns with the pursuit of Sustainable Development Goal 3, especially in terms of mental health, well-being, and substance abuse.

The present findings’ generalisability to all Malaysian undergraduate students may be limited owing to the sample being limited to undergraduate students at UiTM, Puncak Alam. Future research should expand its sample to all university students in Malaysia, considering the validity of the AHLQ and the importance of a healthy lifestyle in mental health. The AHLQ could be translated into various languages to accommodate cultural and environmental differences, potentially yielding valuable findings and discussions. Additionally, future researchers could conduct a CFA to test constructs across different populations. The study did not perform a CFA owing to the questionnaire’s adaptability and the need to identify latent factors within 22 items. A CFA could verify these factors using a priori factors explored in the EFA. An appropriate questionnaire is essential to accurately assess healthy lifestyles relative to mental health among undergraduate students at UiTM, Puncak Alam. Based on the findings, the AHLQ is suitable for use in this population because the items are aligned with the cognitive abilities and characteristics including the cultural background of the students.

## Data sharing statement

All data generated and analysed in the study are available upon reasonable request to the corresponding author.

**How does this paper make a difference in general practice?**

- The adapted scale can be used to identify the most significant components of a healthy lifestyle related to mental health.
- Identifying the most significant components can assist universities in planning strategies, policies and interventions.
- The valid and reliable data obtained from the population can serve as a basis in enhancing the effectiveness of interventions.
